# Steps in the design, development and formative evaluation of obesity prevention-related behavior change trials

**DOI:** 10.1186/1479-5868-6-6

**Published:** 2009-01-21

**Authors:** Tom Baranowski, Ester Cerin, Janice Baranowski

**Affiliations:** 1 USDA/ARS Children's Nutrition Research Center, Department of Pediatrics, Baylor College of Medicine, Houston, TX, USA

## Abstract

Obesity prevention interventions through dietary and physical activity change have generally not been effective. Limitations on possible program effectiveness are herein identified at every step in the mediating variable model, a generic conceptual framework for understanding how interventions may promote behavior change. To minimize these problems, and thereby enhance likely intervention effectiveness, four sequential types of formative studies are proposed: targeted behavior validation, targeted mediator validation, intervention procedure validation, and pilot feasibility intervention. Implementing these studies would establish the relationships at each step in the mediating variable model, thereby maximizing the likelihood that an intervention would work and its effects would be detected. Building consensus among researchers, funding agencies, and journal editors on distinct intervention development studies should avoid identified limitations and move the field forward.

## Background

Obesity is at epidemic proportions in the United States (US)[1] and growing around the world[2]. While there has been a call for increased emphasis on lifestyle factors for obesity prevention[3], there is a crisis in the conduct of community interventions for promoting dietary and physical activity change. Repeated reviews have indicated that most obesity prevention interventions have attained only limited or no behavioral changes; they have rarely impacted the targeted physiological or anthropometric health outcomes; and no common patterns of effect have emerged to differentiate the few successful from unsuccessful programs [4-6]. This situation is not of recent vintage[7]. Also, there is no thoroughly supported evidence-based guidance on what should be done[8]. Many government agencies are experiencing pressures to "act" (i.e. implement community obesity prevention programs) and important efforts have been made to map reasonable courses of community wide action[9]. However, premature action with repeated failures is a waste of public resources and may lead to loss of public confidence in community interventions.

The response of the obesity prevention research community needs to be more carefully planned to build a cumulative science of behavior change and evidence-based guidance[10]. The complexity of influences on adiposity and variations in influences by socioeconomic, and geographic factors have been outlined[9]. Researchers need to systematically develop dietary and physical activity behavior change programs that take this complexity into account by using the best available behavioral, social, and ecological theories and methods[11]. Concurrent with such a shift, funding agencies, their peer reviewers and journal editors should accept clearly defined research study steps in the design, development, and formative evaluation of programs that they are willing to fund and publish. Although several groups have addressed the need for a formative phase in developing interventions[11,12], this paper proposes a series of four formative studies that should be conducted to avoid the method and conceptual problems of earlier efforts, and thereby build a stronger foundation to design effective interventions and more likely detect their effects.

Two large well funded studies were selected to provide examples of top obesity prevention intervention efforts: an elementary school-based study (a very popular intervention channel for reaching children) in a high risk group[13] and a large national media based study (commonly believed to be a channel for large public health benefit)[14]. The school-based project did not achieve intervention related differences in some indicator of body composition, while the media project detected differences across exposure groups.

### Mediating Variable Model

**Figure 1:**
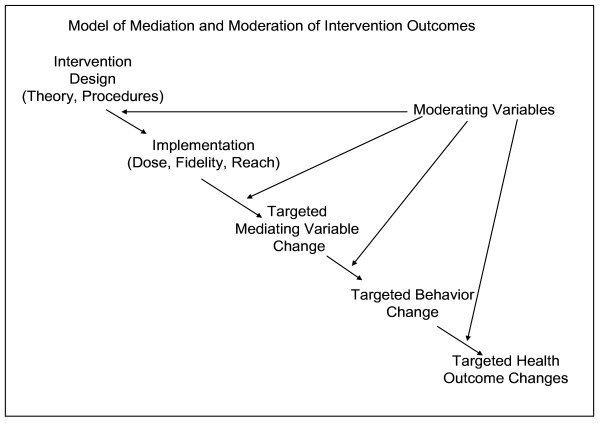
Model of Mediation and Moderation of Intervention Outcomes

The ecological, social, and psychological sciences offer an understanding of why people engage in the behaviors they do. The mediating variable model of behavior change (see Figure 1) posits that intervention programs attain behavior change by inducing changes in mediating variables (that come from the ecological, social, and psychological theories), and changes in these mediating variables induce relatively stable changes in behavior[15] in an approximately linear fashion. Implications of the mediating variable model are that (a) behaviors need to be selected that are maximally and causally related to the health outcomes of concern (or else the health problems will not change); (b) ecological, social and psychological mediators (in the context of known biology) need to be selected that are maximally and causally related to the behavior (otherwise change in mediators will not result in sufficiently large changes in behavior); (c) mediators need to be selected that are highly predictive of the behavior (otherwise substantial changes in the mediators may result in only small or no changes in the behavior); and (d) intervention procedures need to be identified or developed that effectively manipulate the mediators at acceptable levels (or else participants will not receive an effective intervention dose). Problems in previous intervention programs and their evaluations have been identified at each stage and component of the mediating variable model, including a) targeted behaviors were not related to health outcomes in target groups[16]; b) inadequate measurement of the behavior impeded detecting a relationship with adiposity[17,18]; c) hypothesized mediating variables were unrelated to, or even suppressed changes in, the behavior[19]; d) poor quality of the measure of the mediating variable inhibited detecting relationships [20]; e) interventions did not impact mediator(s)[15,21]; and f) inadequate intervention implementation led to failure to detect intervention effects on the mediators[22]. A stepwise approach to designing and developing dietary and physical activity behavior change interventions should minimize these limitations and, thereby, maximize the likelihood of success.

### General Issues in the Design of Behavior Change Intervention

Behavior change interventions must be developed to meet the needs and capitalize on the strengths of specific groups of people (i.e. specific demographic characteristics), using a specific channel (i.e. a delivery method). Thus, a pre-step in designing a behavior change intervention is to select a targeted population (e.g., all 9–11 year old children) using a specific channel (e.g., elementary schools). The choice of channel engages both its strengths and limitations[23,24]. For example, using tribal schools enables reaching large numbers of Native American students, but also encounter layers of approval (and delay) from tribal councils[13]; use of the larger media can reach large numbers of citizens nationwide, but could involve inconsistent messaging when developed by media writers, imposes difficulties in measuring heights and weights from participants, and makes control groups and randomization practically impossible[14].

Adiposity indicators (e.g., BMI, waist circumference, skinfolds, waist to height ratio[25]) must be selected carefully because a) different program outcomes could be obtained with different indicators[26]; b) in some populations the selected indicator and adiposity do not seem to be interrelated as expected[27,28]; and c) the indicators differentially relate to key socio-demographic factors[29]. Raw BMI (as opposed to gender and age specific centiles or z scores) may be the best metric when assessing changes[30]. One of the example programs generated a population specific multiple indicator regression equation to predict percent body fat[31] to minimize the limitations of using BMI alone[27]. This should provide a model for others to consider. The other[14], however, used self reported height and weight which has severe problems with accuracy. Inadequate measures, even with large samples, make it difficult to detect effects, and may even lead to erroneous conclusions[32].

### Formative Step A. Targeted Behavior Validation

Behaviors should be targeted for change that are causally and substantially related to the health problem. While some behaviors are clearly related to a health problem (e.g., cigarette smoking and lung cancer), obesity does not have clearly empirically verified universal behavioral causes[16]. For example, the literature has been reviewed on the relationship of sweetened beverage consumption to obesity[16]. Limitations in the research methods to date would not permit definite conclusions about the relationship[16]. At one time dietary fat intake was considered the primary cause of obesity [33], but that perception has changed as well[34]. The behavior to health outcome relationship may exist in certain groups (e.g., elementary school aged children), but not the one targeted for intervention (e.g., pre-school children[35]). Single nutrients or food groups thought to be predictive of obesity (e.g. sucrose) may simply be indicative of an overall poor diet quality[36]. People tend to eat multiple foods organized into consistent patterns overtime[37]. There may be value in targeting patterns of dietary intake, rather than specific nutrients or foods, since these may be more strongly related to the health outcomes[38]. However, even here, the relationships in certain groups don't always emerge as expected (e.g., the prudent diet predisposed to breast cancer among women in New Mexico[39], and the vegetable rich pattern was associated with obesity among the Chinese[40]). The place where people eat (e.g., restaurants, friends' homes) may be a marker of poorer dietary practices and provide a useful intervention target[41].

Similar problems exist in regard to selection of a targeted level of physical activity. Although 60 min of moderate to vigorous physical activity per day 3 to 5 days per week has been prescribed as the desirable level to prevent obesity and other chronic illnesses[42], children engaging in that level (or more) over a three year period (using an objective measure) were not leaner than children who did not[43]. There have been disagreements about whether one should measure physical activity or fitness[44], and concerns about the quality of measurement especially when using self-reports[45]. One of our example programs detected change in self-reported, but not objectively assessed, physical activity, suggesting some self-report bias[13].

There have been a number of efforts to identify the factors accounting for the obesity epidemic[16,46], with no clear strong consistent findings. Poor diet and physical inactivity may not be primary causes of the current obesity epidemic[47]. Lack of self control of satiety (not specific foods)[48] may be a major contributor. Alternatively, obesity appears likely influenced by a multitude of neither sufficient nor necessary factors that interact and sometimes trigger compensatory behaviors. For example, lower energy intake from specific foods may be compensated by an equal increase of energy intake from other nutrients and foods[49], or increases in energy expenditure[50]. The fact that very large samples (hundreds of thousands) are needed to detect biologically plausible dietary and physical activity behavior to obesity relationships[51] likely indicates a) compensatory behaviors, b) heterogeneity of effects across population subgroups, and c) poor quality of measures of behavior and health outcomes. One example program using more objective observational measures of intake at school lunch detected differences in fat intake, but not total energy, suggesting compensation for energy dense fat with other foods[13].

Research clearly delineating the causes of obesity may be the most important contribution to obesity prevention behavior change research[52] at this time. Formative Step A research should assess the metric qualities (reliability, validity) of the methods employed in their hands; identify possible confounders and moderators of cross-sectional or, optimally, longitudinal behavior-outcome relationships (e.g., physical activity moderating a caloric intake to adiposity relationship); and include measures of common response biases (e.g social desirability of response).

A realistic assessment must be made of the extent of change in caloric imbalance necessary to redress the obesity problem in the target population, and thereby the number and extent of behavior changes required[53]. To minimize the likelihood of selecting ineffective behaviors to change in intervention, the possible behavioral influences on obesity should be identified, and one or more selected for change that are most strongly related to obesity; while paying attention to possible compensatory effects, with some evidence that a) the behaviors are amendable to change; b) there are known models for why people do those behaviors (sources of mediating variables); and c) changes in the behaviors can be reasonably easily and precisely measured to evaluate the outcome.

Investigators need to address how the intervention channel (e.g., the elementary school) influences the behavior (e.g., foods offered in school breakfast, school lunch, alternative food line, vending machines), the environment of the channels (e.g., proximity of the schools to fast food stores), and especially the behaviors performed/exhibited in those environments. Otherwise promising interventions may fail because they ignored channel and channel-environment effects[23].

Often the literature is not replete with findings relating specific behaviors to obesity in the group targeted for change, so empirical support needs to be generated. Under these circumstances, research should be conducted measuring both the behavior and the selected adiposity indicator(s) in the targeted group in the selected channel. Preliminary qualitative research should be conducted (using focus groups or intensive interviews) of what and how the behaviors are performed in the selected channel (e.g., selection of alternative foods (snack bars) in school lunch in elementary schools), nuances in their performance which may influence mode of measurement (e.g., frequent exchanges of foods between those bringing food from home and those obtaining it at school, which means one can't simply ask a parent what their child ate at school) and cognitive interviews with participants in how they understand the items measuring the behaviors (if some form of self-report is the method selected). The research must clarify whether the primary contributions to adiposity are more likely to occur at school (or at work for adults) or at home[54].

Acceptable levels of quality of measurement and expected relationships should be pre-specified as decision making stop criteria prior to the study. For example, appreciable levels of misclassification error occur at levels of validity below 0.90[55]. While this level is not achievable with current assessment methods, especially with respect to diet and physical activity, there are lower levels of validity at which it would be virtually impossible to detect relationships, or be sensitive to intervention outcomes[56].

Conducting such a study will have the beneficial effects of creating recruitment, measurement and training protocols, demonstrating access to the target population (the ability to recruit a sample), and enabling assessment of possible participation and response biases in collecting such data. Sample descriptive statistics will become known upon which sample size calculations can be made to adequately statistically power an outcome evaluation[57]. Finally, some estimate will be obtained of the level of relationship of the behavior to adiposity, which places an obvious limit on how much change in the adiposity could be obtained in an intervention targeting that behavior. The lack of such a relationship may either suggest it does not exist, or cannot be detected with the methods employed in the targeted group. Either interpretation would question whether the investigators should progress in developing the intervention. Less than acceptable performance on the set criteria of measurement quality will require this Type A study to be improved and repeated, or force the investigators to rethink the target group, target behaviors, and/or data collection procedures.

Funding agencies should be willing to fund such formative research that provides the foundation for an intervention from clearly specified research grant mechanisms.

### Formative Step B. Targeted Mediator Validation

With a successful Step A, the investigators must now select the demographic, ecological, social, psychological, and biological variables which will become the mediating and moderating variables for the intervention. One or more behavioral theories should be selected to guide the development of intervention procedures. Particular attention should be paid to documented moderation of mediator-behavior relationships and inconsistent mediation effects (e.g., the presence of competing mechanisms with opposite effects on the outcome)[58]. A balance must be drawn between influences on the individual to change their patterns of behavior, and to change the environment to support (and not subvert) those behavior changes[59], with the possibility of stratifying or exclusively focusing on a specific ethnic group[59], gender, biological influences (e.g., genes[60] or aspects of early growth[61]), or group of individuals with specific environmental, psychosocial, and outcome baseline characteristics[62].

While no one intervention can address all possible influences, the investigative team must select those that theoretically and empirically appear to be the most influential variables for which there are known likely to be effective methods for changing them. In both of our example studies, it is not clear how the intervention specifically capitalized on behavioral theory to promote change in the behaviors[13,14]. Thus, a conceptual model[63] should be developed that clearly specifies how these influences interrelate and relate to the targeted behavior(s). This should include available estimates of effect sizes for the associations between the mediating variable and the behaviors[64]. Priority should be given to polytheoretical models to maximize predictiveness (especially since this is not meant to be elegant theory testing research), thereby obtaining the biggest handles for changing behavior[65]. The available research should optimally adopt a longitudinal design to explore relationships between changes in mediators and changes in behavior, as cross-sectional and longitudinal associations may differ substantially[66]. A longitudinal research design would also allow testing for temporal stability and for possible exposure effects on interpretation of the survey items[67]. For each of the selected influences, measures must be specified that have been used in the target population with acceptable levels of psychometric characteristics and shown to be predictive of the targeted behavior at levels high enough to expect that change in the mediator will lead to change in the behavior. If such measures have not clearly been validated in comparable target populations, cognitive interviews would be beneficial to assess the target groups' understanding of the items[68].

Most often, quantitative studies have not been conducted of the major predictors of the targeted behavior with the targeted group in the targeted channel. Thereby, new data should be collected starting with preliminary formative qualitative research (e.g., focus groups, intensive interviews).

A sufficiently large sample is necessary to assess the psychometric characteristics of the instruments and to assess the fit of the conceptual model to the data. Preferred methods require that the sample be large enough to conduct the desired analyses in both exploratory and confirmatory subsamples. Investigators should be considering samples of at least 400 to 500 participants, determined in large part by the requirements of the analyses proposed. Optimally, the predictiveness of the model across sub-groups of possible participants should be assessed to verify the need for different approaches to intervention (identification of moderators). Obviously, this would require even larger samples.

The outcomes of this research step will be validation (or need for further validation) of the selected measures of mediating variables; known predictiveness of the selected model with an understanding of which variables are most highly predictive or otherwise centrally involved, and thereby deserving priority in intervention design; and partial information necessary to estimate the sample size to detect mediated effects in the efficacy evaluation.

One of the example studies used measures of knowledge (which have rarely been related to behavior change) with inadequate levels of reliability (rel < 0.55)[13]. Smaller changes in third grade as opposed to 4^th ^or 5^th ^grade knowledge may have been a function of unreliable measurement. The other example study provided no psychometric characteristics on any of the self-reported measures employed[14,69].

The research team should pre-specify acceptable levels of psychometric characteristics and of levels of predictiveness (e.g., if investigators cannot account for at least 25% of the variance in the behavior, or changes in the mediator yield reliable but trivial changes in the behavior, it is probably not worth proceeding to intervention). Funding agencies should fund Step B projects from funding mechanisms clearly specified for this purpose. This step may need to be repeated if the desired psychometric characteristics or level of predictiveness are not obtained.

### Formative Step C. Intervention Procedure Validation

With the clearly specified model, the investigators must identify which mediating variables they will prioritize to change and identify procedures that maximize the likelihood and extent of doing so (i.e. formulate their action theory)[63]. Identifying empirically validated effective mediating variable change procedures may be the hardest issue to find addressed in the published literature. For example, these issues are just now being addressed in the addictive substances literature, and they cannot find patterns in successful versus unsuccessful studies[70]. Most investigators have either used an intuitive approach to specifying how they would change mediators, or post-dicted it, i.e. assessed whether intervention procedures changed the variables they selected for mediators after the intervention was designed (probably on an intuitive basis)[71]. This is not acceptable since it risks capitalizing on chance. The field would benefit from a clearly articulated theory-based taxonomy of change procedures[72] and thorough empirical base evaluating each in diverse demographic groups and channels.

Most investigators will want to focus on several change procedures, at least one for each of the mediating variables, or otherwise the development process will be interminable. The investigators will need a protocol to specify how staff should implement each change procedure, a training manual with certification procedures, and a quality review manual to periodically assess if it is being done as specified for each procedure studied. These manuals should be based on the best understanding of how to get professionals to change their practices, including high specificity[73].

If the literature clearly delineates one or more procedures that would attain acceptable levels of change in the selected mediating variables in the targeted population and channel, and this level meets investigator needs, then the investigator may proceed to Step D. In most cases, however, the investigator will need to design one or more procedures, based on the knowledge of the theory and its mediating variables and of other change procedures (e.g., persuasive messages[74], skill development[75]), and test their effects on the targeted mediating variable(s) in the targeted sample and channel. Elsewhere, this has been called evidentiary research[26]. The investigators should pre-specify a level of change they are willing to accept to progress to the next step for each targeted mediating variable, and below which they are not (because it would compromise the efficacy study). If in step C moderating variables are identified, the investigators need to devise and test separate procedures for each of the subgroups.

The evaluation should include both quantitative measures of the targeted mediating variables and qualitative interviews that assess what participants perceived to be acceptable and unacceptable about the procedure(s), and suggestions for improvement. Funding agencies should fund step C studies from mechanisms clearly specified for this purpose. This step may need to be repeated if the desired level of change is not attained. If no clear refinements of change procedures are available, the investigator may wish to select other mediating variable(s) (with procedures with a likely acceptable level of change) on which to repeat tests of single component intervention procedures. Some investigators with small research capabilities may wish to become purveyors of Step C research, which would still be a substantial contribution to a corpus of intervention research. Funding mechanisms specific to Type C research are to be generated. One of our example studies placed heavy emphasis on increasing knowledge[13], even though a previous thorough review of the literature indicated knowledge change was not related to behavior[7]. The school-based intervention study[13] used procedures that were not demonstrated to influence the corresponding behaviors in the targeted population which has rarely been studied. The evaluation of the media intervention[69] could not differentiate which of the many intervention components contributed to change.

### Step D. Pilot/Feasibility Intervention

At this stage, investigators should have a clear idea of what intervention procedures they will use to target each selected mediating variable, along with the associated intervention protocols, staff training procedures, and quality control of implementation procedures. The investigators will also need to address issues in combining the procedures, e.g., sequencing, attaining synergies, and appropriate efficient use of staff and other resources. There has been recent concern that interventions were not delivered as designed (posing problems in evaluating program effects[76]), and that the processes have not been adequately reported [77].

Pilot study evaluation should focus on participation bias (i.e., to whom the intervention appeals), feasibility and process evaluation, ensuring that and assessing whether an adequate dose of intervention with a high enough quality was delivered to an acceptable number of participants (reach)[26]. One of the example studies targeted manual laborers, but more non-manual than manual respondents reported hearing of and seeing the program[14].

A qualitative process evaluation should assess participants' perceptions of what went right, what went wrong, and how it might be improved. Investigators should preset criteria for acceptable process evaluation to enable them to make a decision about whether to proceed to the next step. The pilot study should be long enough for staff to experience the challenges of implementing procedures, and for participants to experience it enough to form opinions, but not necessarily for the full duration in an efficacy study. The process evaluation should be used to further refine the procedures and develop new procedures to address unanticipated problems. One of our example studies recently demonstrated progressively improved compliance with food service guidelines over three years (from 51% to 80% to 87%), but only 56% of schools offering 5 PE sessions per week, and 58% average student attendance at family events[13]. In the other only 17 of 1894 respondents (less than 1%) to a random sample survey sent for a registration pack of intervention materials and only 3 of these actually sent it in[14]. Even the most efficacious interventions will have little effect if inadequately delivered.

Some investigators believe that a single intervention may not be adequate to meet the needs of all possible participants (a "one size fits all" intervention[67]). Some participants may need extra dose, or different types of dose, at certain points in the intervention, while highly motivated others may need little more than encouragement or guidance. This attempt at developing an intervention to meet these diverse needs in systematic ways has been termed a branched logic or stepped intervention[78]. The result of step D could be sufficient qualitative with some quantitative data to design such a branched logic intervention. If new procedures are needed, the investigators may need to repeat a Step C study with the subgroup. This option would require an ensuing pilot study to pilot test the implementation of the branched processes before moving on to a Multiphase Optimization STrategy (MOST) program evaluation[78].

### Brief Comments on Intervention Efficacy and Effectiveness Trials

At this point the investigators will have protocols for and substantial experience in all implementation, intervention, and measurement procedures. An efficacy trial is the next step. The efficacy trial tests if a theory-based intervention will work under ideal circumstances (i.e., when adequate resources are available to deliver the intervention exactly as designed with adequate time to deliver it). Efficacy trials have been discussed at length[11,79,80], but a few issues should be pointed out.

A randomized clinical trial is the obvious preferred research design for an efficacy trial. Since science will benefit from understanding both what procedures worked and the corresponding processes of change[81], an appropriate evaluation will include both a process evaluation and a mediating variable analysis[82]. Targeted mediated and outcome variables should be measured as frequently as possible to assess change, their time course, and to relate change in mediators to both delivered dose and change in outcomes[82]. A qualitative process evaluation should assess participants' perceptions of the acceptability of each intervention component, and how it might be improved. Evaluation of a MOST trial would require even more complex evaluation procedures[78,83]. Cost effectiveness studies are inappropriate at this step because the trial was not designed with cost effectiveness considerations in mind. If the intervention does not work in efficacy circumstances, it is very unlikely to work under other less carefully formulated circumstances and so no further studies should be contemplated.

There is an emerging issue that self-report measures could be influenced by repeated use in the same group, or by participating in an intervention[26,84]. This would be the first level of study where these issues could be clearly addressed. Efficacy trials should be reported using CONSORT[85], or other trial reporting protocols[86,87], to assure their optimal contribution to the literature.

Some large scale "efficacy" type intervention trials have changed intervention procedures in the midst of the intervention trial, as some procedures did not seem to work, and/or others appeared to offer more promise[26]. While this may make some practical sense to not lose the benefit of an expensive intervention, it makes interpreting the experiment challenging. Conducting studies A through D with possible repeats to work out the "bugs", should minimize the urge to change intervention procedures in midstream, and thereby provide clearer tests of intervention outcomes.

Most programs in the non-research world do not have the resources (e.g., adequate number of staff with all necessary expertise, adequate resources, sufficient time) to deliver interventions under efficacy circumstances. An effectiveness trial tests whether the principles learned from successful efficacy trials can be implemented to good effect under more "real world" circumstances. A number of investigators have addressed the design of effectiveness type studies [88-90]. Cost effectiveness analyses make most sense when conducted with effectiveness trials, because issues of cost will determine if the intervention (or components thereof) will be disseminated. The CONSORT or similar reporting protocols should be used for reporting effectiveness studies, as well.

An assumption of the proposed approach is that the mediating variable model is valid in accounting for how interventions work. The key assumption is that changes in mediators account for relatively stable changes in behaviors in an approximately linear fashion. Alternative models would be the incentive model[91], i.e. change occurs only in response to tangible incentives; a tipping point model[92], where there is some minimally necessary level of the mediating variable, before which no behavior change occurs; or an activation model, i.e. people with high level of a mediating variable that is "dormant" or "unattended to" have that variable "activated"[93], while only those with low levels of the mediator require its change. (These are all examples of nonlinear or unstable mediating variable relationships.). There do not appear to be enough behavior therapists in the world to manage the contingent delivery of incentives to broadly deliver the incentive model. Little is known about how to activate mediating variables, or measure this dual activation and mediation process. But, if necessary, this could be an area for future research. An issue deserving more attention in mounting such efforts is the organizational structure, number and expertise of staff, linkages to the target group(s) and channels, and many other leadership, infrastructure, and staff motivation issues[94].

The main drawbacks associated with this approach to program development include the extended time and resources needed to complete the various steps in the process. While true, not following a logical sequence of actions that identifies the best available behavioral, social, and ecological theories and methods to tackle obesity in particular populations and settings, we maximize the risk of wasting time and resources. The current state of affairs is testimony to these concerns.

## Conclusion

Intervention researchers and practitioners need to engage in a process to design and develop interventions that maximize their programs' likely effects and what can be learned from such trials. Funding agencies also need to have a clear idea of a process for program development to know how to support these important activities. This manuscript proposes such a process. Further discussion should be stimulated among the various interested parties, and hopefully a consensus will emerge on the optimal processes in which to engage and support.

## Abbreviations

BMI: Body Mass Index; MOST: Multiple phase Optimization STrategy, a type of program evaluation; CONSORT: CONsolidated Standards Of Reporting Trials.

## Competing interests

Tom Baranowski serves on the Global Advisory Committee for the McDonald's Corporation. The authors have no other competing interests.

## Authors' contributions

TB wrote a first draft of this manuscript. EC made numerous contributions to ensuing drafts. JB made intellectual contributions throughout the development of this manuscript, and edited a near final draft.
